# An Enhanced Technique for Ultrasonic Flow Metering Featuring Very Low Jitter and Offset

**DOI:** 10.3390/s16071008

**Published:** 2016-06-29

**Authors:** Assia Hamouda, Otto Manck, Mohamed Lamine Hafiane, Nour-Eddine Bouguechal

**Affiliations:** 1 Institut für Technische Informatik und Mikroelektronik, Technische Universität Berlin, Einsteinufer 17, Berlin 10587, Germany; manck@mikro.ee.tu-berlin.de (O.M.); hafiane_lamine@mikro.ee.tu-berlin.de (M.L.H.); 2 Laboratoire d’Electronique Avancée, Département d’Electronique, Université Batna 2, Rue Chahid Boukhlouf, Batna 05000, Algeria; bougnour@univ-batna.dz

**Keywords:** ultrasonic water flow meter, jitter, transit time difference (TTD), least-square-sine-fitting

## Abstract

This paper proposes a new, improved method for water flow metering. It applies to a transit time ultrasonic flow meter device. In principle, the flow of a given liquid in a pipe is obtained by measuring the transit times of an ultrasonic wave in the upstream and downstream directions. The difference between these times is, in theory, linearly proportional to the liquid flow velocity. However, the fainter the flow is, the smaller the transit time difference (TTD) is. This difference can be as low as a few picoseconds, which gives rise to many technical difficulties in measuring such a small time difference with a given accuracy. The proposed method relies on measuring the TTD indirectly by computing the phase difference between the steady-state parts of the received signals in the upstream and downstream directions and by using a least-square-sine-fitting technique. This reduces the effect of the jitter noise and the offset, which limit measurement precision at very low flow velocity. The obtained measurement results illustrate the robustness of the proposed method, as we measure the TTD at no-flow conditions, with a precision as low as 10 ps peak-to-peak and a TTD offset of zero, within a temperature range from room temperature to 80 °C. This allows us to reach a smaller minimum detectable flow when compared with previous techniques. The proposed method exhibits a better trade-off between measurement accuracy and system complexity. It can be completely integrated in an ASIC (application specific integrated circuit) or incorporated in a CPU- or micro-controller-based system.

## 1. Introduction

Water flow measurement is often critical in the domestic sector of the economy [1]. Therefore, it is imperative to accurately measure what flows through the pipe under all circumstances, in order to make profit rather than run at a loss. Ultrasonic flow meters have been working successfully in industrial applications for several decades. They have gained approbation in wide metering applications such as dirty or clean water, petrochemical products, and natural gas. This is due to significant operational and economic advantages that the ultrasonic flow meters offer in contrast to conventional meters. Economically, they are easy to install, inexpensive, and require less maintenance than other meters such as mechanical flow meters, which need to be checked periodically. Operationally ultrasonic flow meters can be highly sensitive and accurate, and they typically have a broader flow rate range, good measurement repeatability, and bi-directional flow capability [2,3].

**Figure 1:**
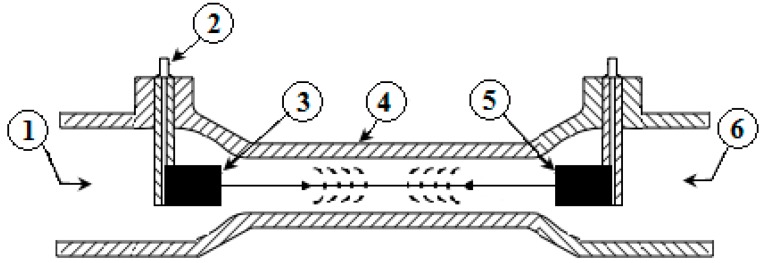
Flow meter pipe with in-line transducers: (1) inlet; (2) cable connector; (3) upstream transducer; (4) flow meter body; (5) downstream transducer; (6) outlet.

The basic geometry of a transit time ultrasonic flow meter considered in this work consists of two transducers mounted coaxially, as depicted in Figure 1. The transducers (3 & 5) are separated by a known distance L and face one another such that an ultrasonic signal can be sent from either one and its arrival detected by the other. This signal is generated by the piezoelectric crystal when it is subjected to an alternating voltage; conversely, the piezoelectric crystal generates a voltage when the ultrasonic signal impacts the transducer. The ultrasonic signal can travel through the pipe in the direction of the flow (downstream direction) and against the flow direction (upstream direction). It travels faster in the direction of the flow and slower against the flow. The difference between the two transit times is directly proportional to the flow velocity. This can be mathematically expressed as follows (see Section 2 for more details):

(1) $$v=\frac{c^{2}\Delta t}{2L}$$

where Δt is the difference between the upstream and downstream traveling times t_up_ and t_down_, L is the effective length of the travelling-path of the ultrasonic waves (see Figure 1), and c is the propagation speed of the sound in a given liquid (water in our application).

When there is no flow in the measuring pipe (still water), the upstream and downstream transit times of such meters are the same and the difference between the two transit times should be negligible. This may not be the case since the transit time may be influenced by jitter and offset. Therefore, any delay offset between the upstream and downstream transit times translates directly to a zero-flow error. This zero-flow offset limits measurement accuracy at low flow velocities. The smaller this error is, the more accurate the flow meter will be.

Several approaches have been proposed in the literature to detect accurately the transit time difference (TTD) of an ultrasonic flow meter. The zero-crossing method is commonly used and consists of detecting the zero-crossings of the received signals in the upstream and downstream directions [4]. These zero-crossings are compared with the corresponding zero-crossings of the transmitted signals in the upstream and downstream directions, and the difference is taken to be the transit time. Based on this method, Texas Instruments reported [5] an 80 ps peak-to-peak jitter and TTD-offset drift of about 25 ps measured at no-flow conditions.

Yang et al. reported [6] an approximately 200 ps peak-to-peak jitter and −185.3 ps TTD-offset drift measured at no-flow conditions. Their approach consisted of driving both transducers at a specific frequency outside their resonance frequencies in order to eliminate the effect of temperature dependence of the resonance frequency. However, the main drawback of this technique is the degradation of the signal-to-noise ratio (SNR), caused by working outside the resonance frequency of the transducers.

As mentioned before, the main challenge facing the ultrasonic transit time flow meters is to measure the TTD in the picosecond range. In our approach, this is achieved by deducing the TTD from the phase difference, with a sine wave excitation, between the upstream and downstream received signals. The phase difference is estimated using the least-square-sine-fitting (LSSF) method, which is a very accurate method to estimate all parameters (amplitude, phase, and frequency) that characterize a digitized sinusoidal signal, sampled at a well-defined sampling rate [7,8].

This paper presents a new approach to measure accurately the water flow based on ultrasonic devices. We describe first the methodology employed to compute the TTD. We then investigate the impact of the jitter noise on the measurement accuracy and how to reduce the jitter and the TTD-offset drift using the proposed method. We analyze the obtained measurement results and compare them with the theoretical background.

## 2. Transit Time Flow Meter Principle

The ultrasonic signals travel through the pipe in the direction of the flow (downstream direction) and against the flow (upstream direction), during a time interval called transit time, in order to be received by the opposite transducer. Assuming v is the flow velocity in the pipe, the upstream and downstream transit times t_up_ and t_down_ are given by [9,10]

(2) $$t_{\mathrm{up}}=\frac{L}{c-v}$$

(3) $$t_{\mathrm{down}}=\frac{L}{c+v}$$

Thus,

(4) $$\mathrm{TTD}=\Delta t=t_{\mathrm{up}}-t_{\mathrm{down}}=\frac{2\mathrm{vL}}{c^{2}-v^{2}}$$

Assuming $v^{2}\ll c^{2}$ (flow velocity v is much smaller than the speed of sound c),

(5) $$\Delta t=t_{\mathrm{up}}-t_{\mathrm{down}}=\frac{2\mathrm{vL}}{c^{2}}$$

hence,

(6) $$v\;\approx \frac{c^{2}\Delta t}{2L}$$

When there is no-flow in the pipe,

(7) $$t_{\mathrm{up}}=t_{\mathrm{down}}=t_{0}=\frac{L}{c}$$

The ratio of Δt to the traveling time t_0_ measured at no-flow conditions is given by

(8) $$\frac{\Delta t}{t_{0}}=\frac{2v}{c}$$

Equation (8) can be used to calculate the needed measurement accuracy. For instance, the minimum flow velocity v_min_ calculated for a given flow rate of two liters per hour (2 l/h), and a pipe diameter of 0.8 cm is about v_min_ = 10 mm/s. Therefore, according to Equation (5), this minimum flow velocity of 10 mm/s produces a TTD value of about Δt = 380 ps, provided that the travelling time of the used flow meter pipe is 28 µs (calculated for L = 42.2 mm using Equation (7)). Therefore, to achieve an accuracy of 5%, the desired ultrasonic flow meter must be able to measure accurately a minimum transit time difference value of at least 20 ps.

## 3. System Description

**Figure 2:**
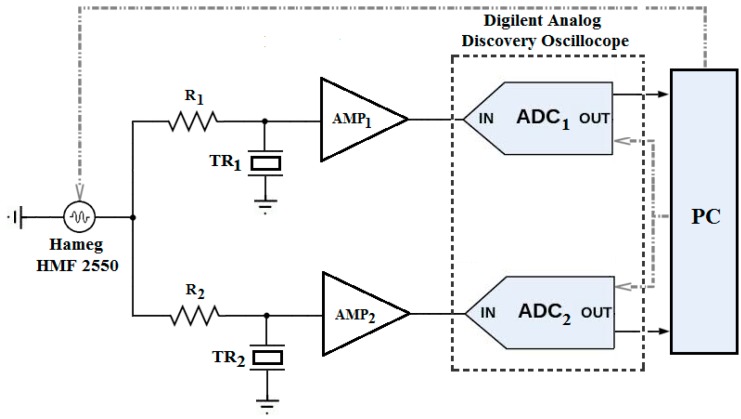
Diagram of the experimental system.

The principle of operation of the system used in the experiments is illustrated in Figure 2. In order to allow the receiving transducer to reach a steady state, where both amplitude and frequency are settled, a sinusoidal burst of 70 cycles generated over a time period of 100 ms by a function waveform generator Hameg HMF 2550 (Rohde & Schwarz Co. KG, Munich, Germany) is used to drive the two transducers (TR_1_ and TR_2_) simultaneously through the resistances R_1_ and R_2_. These transducers convert the electrical excitation into a mechanical wave. The driving frequency of the burst is selected near the transducers resonance frequencies so that the transducers achieve maximum output vibration with an enhanced SNR.

**Figure 3:**
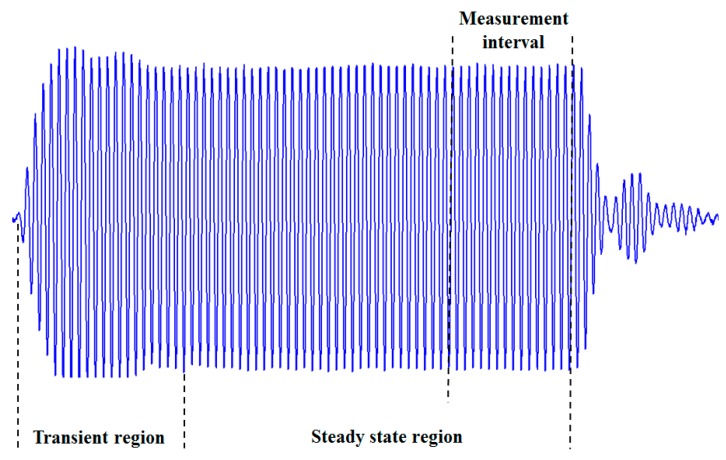
Response of the 4 MHz transducer to the incident ultrasonic wave.

The TTD can be deduced from the phase difference between the steady-state parts of the received signals in the upstream and downstream directions, where the estimation of the two phases can be performed using the least-square-sine-fitting algorithm of Matlab.

(9) $$\varphi_{\mathrm{up}}=2\pi {\mathrm{ft}}_{\mathrm{up}}$$

(10) $$\varphi_{\mathrm{down}}=2\pi {\mathrm{ft}}_{\mathrm{down}}$$

where $\varphi_{\mathrm{up}}$ and $\varphi_{\mathrm{down}}$ are the phase shifts measured during the steady-state regions (Figure 3) in the upstream and downstream direction respectively. In the steady-state region, the received signal frequency f matches the forced frequency (the driving frequency of the exciting signal). Therefore, the TTD can be computed from

(11) $$\varphi_{\mathrm{up}}-\varphi_{\mathrm{down}}=2\pi f\left(t_{\mathrm{up}}-t_{\mathrm{down}}\right)$$

In order to eliminate the high starting jitter, caused by triggering and recording the ultrasonic signals in both directions separately (sequentially), we rely on the simultaneous (synchronous) excitation approach. This means that the common synchronized clock of the digital oscilloscope (Digilent Analog Discovery) [11] is used to trigger and acquire the signals in both directions. Hence, we cancel any differential delay time between the two input channels.

**Figure 4:**
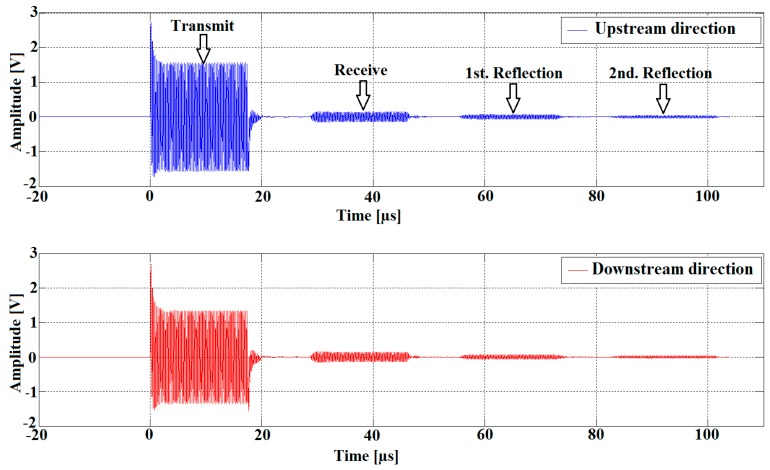
Acquired ultrasonic signals in the upstream and downstream directions (measured using a driving frequency of 4 MHz).

As shown in Figure 4, the sound waves are received simultaneously by both transducers after a traveling time proportional to the distance between the transducers. The received signals are attenuated due to the losses and the absorption of acoustical energy by the medium [12]. Therefore, we use two low noise operational amplifiers OPA2846ID (Dallas Semiconductor Inc, Dallas, TX, USA), as shown in Figure 2, in order to increase the signal levels and thus improve the SNR. The amplified signals are acquired by the digital oscilloscope, which uses dual-channel, 14-bit, ADCs with a sampling rate of 50 MS/s. The samples are saved in CSV data format and imported to the PC in order to compute the TTD. An embedded (PC) software algorithm written in Matlab handles the automatic TTD measurement repetition, providing all the control subprograms for the control of the function generator and digital oscilloscope.

## 4. Effect of Jitter on TTD

**Figure 5:**
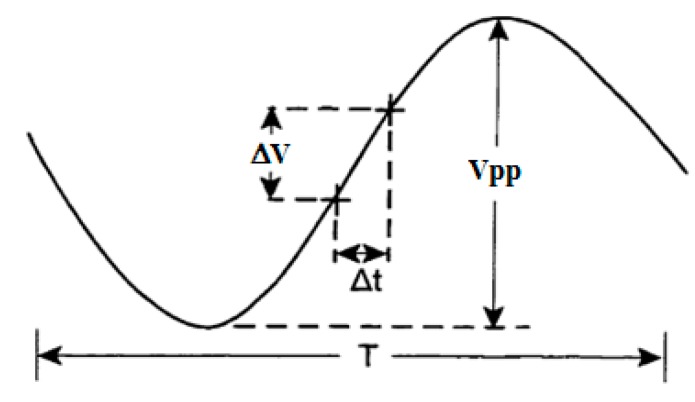
Effect of the sampling jitter.

Jitter is the dispersion of the measured TTD around the mean value (zero for no-flow). Commonly, it refers to the random errors in the time location of the waveform samples. The latter introduces corresponding errors in the amplitude value. As depicted in Figure 5, the jitter can be defined as a deviation of the sampling instant of a given signal from their ideal location in time (x-axis) influencing proportionally the y-axis (amplitude of the signal). Concerning our application, for an assumed sine wave (ultrasonic waveform in the steady-state) of period T and peak-to-peak amplitude Vpp, a sampling time jitter Δt, near t = 0, will cause a change in the measured value equal to ΔV = Vpp π Δt/T [13].

**Figure 6:**
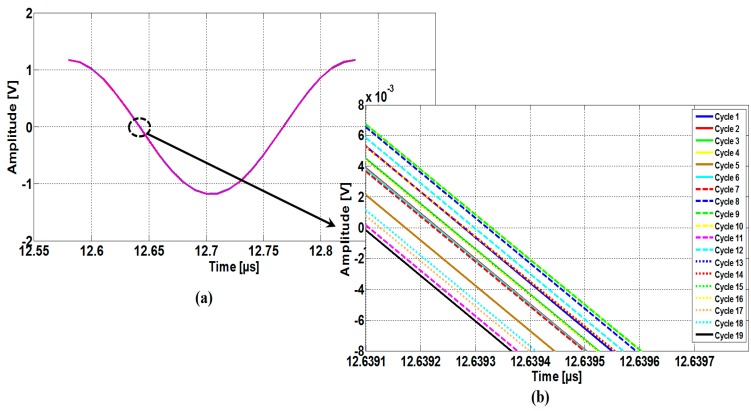
Cycle-to-cycle jitter around zero-crossing: (**a**) last 19 periods of the transmitted signal superimposed upon each other; (**b**) Magnified view around zero-crossing.

Typically, the transducers are excited by a sinus burst of 70 cycles at a frequency of 4 MHz. The experimental results, reported in Figure 6, presents the eye diagram of the last 19 periods of the transmitted signal superimposed upon each other. Figure 6b shows a magnification of Figure 6a. The average of T, in the steady-state region, is 250 µs, whereas the cycle-to-cycle jitter noise is about 200 ps (peak-to-peak). This deviation leads to an amplitude fluctuation of about 7 mV.

This jitter can be explained by the fact that the transducer structure consists of small crystallites or grains [14]. When the latter are excited periodically by an external burst of a finite number of cycles, there is a remaining irregular stress between these small crystallites, which is expected to occur during the next oscillation of the transducer. Moreover, the signal acquisition path contains many additional jitter sources, such as jitter in the sampling time that may occur due to imperfect hold circuit synchronization, quantization error of the ADC, and cross-talk between the cables [7].

## 5. Jitter Reduction Technique

Since the recorded data of the received waveform are sampled with a sampling rate of 50 MS/s, each period in the steady-state region contains around 13 sample points. The sine-fitting approach adjusts Equation (12) to the set of recorded sampling data in order to extract the characterized parameters of the trace, namely, the amplitude, the phase and the frequency.

(12) $$x\left(n\right)=\mathrm{Asin}\left(2\pi {\mathrm{ft}}_{n}+\varphi \right)$$

where A and φ are the amplitude and the phase of signal, respectively, f is the driving frequency, and t_n_ is the discrete-time vector.

The following equation, obtained from Equation (11), is used to compute the TTD from the upstream and downstream extracted phases ($\varphi_{\mathrm{up}}$, $\varphi_{\mathrm{down}})$:

(13) $$\mathrm{TTD}=\frac{\varphi_{\mathrm{up}}-\varphi_{\mathrm{down}}}{2\pi f}$$

The precision with which the TTD is determined can be influenced by two parameters: the amplitude of the received signal and the number of the samples used in the fitting algorithm. Concerning the first parameter, there is a clear correlation between the jitter level and the amplitude of the received signal in the steady-state region. In other terms, the obtained TTD has a jitter that depends on the input dynamic range of the ADC. This is optimized through the adjustment of the gain of the two amplifiers (Figure 2), aiming to cover most of the input range of the ADC. The ADC dynamic range, which is the ratio of the maximum voltage V_FRS_ to the minimum voltage V_LSB_, can be expressed as the number of bits or the resolution.

(14) $$N_{\mathrm{bit}}=\frac{\log\left(\frac{V_{\mathrm{FSR}}}{V_{\mathrm{LSB}}}\right)}{\log2}$$

**Figure 7:**
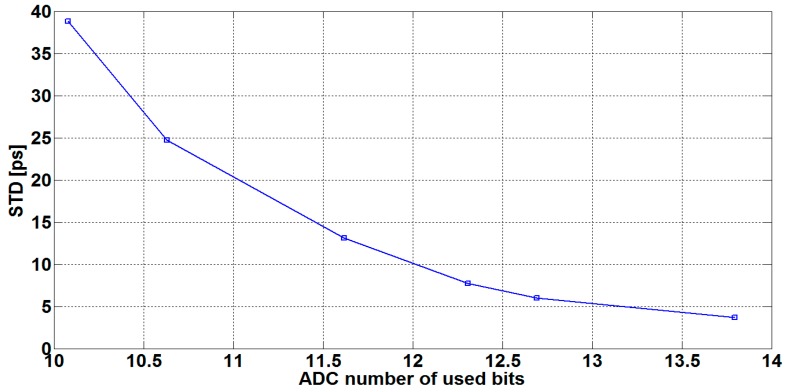
Transit time difference (TTD) standard deviation versus number of ADC bits (N = 250).

Figure 7 illustrates the jitter measurement precision (represented by the standard deviation) versus the number of the ADC used bits. The standard deviations are calculated from TTDs of 50 captured ultrasonic waveforms. The measurements were carried out at room temperature at no-flow conditions, and N = 250 fitted samples.

**Figure 8:**
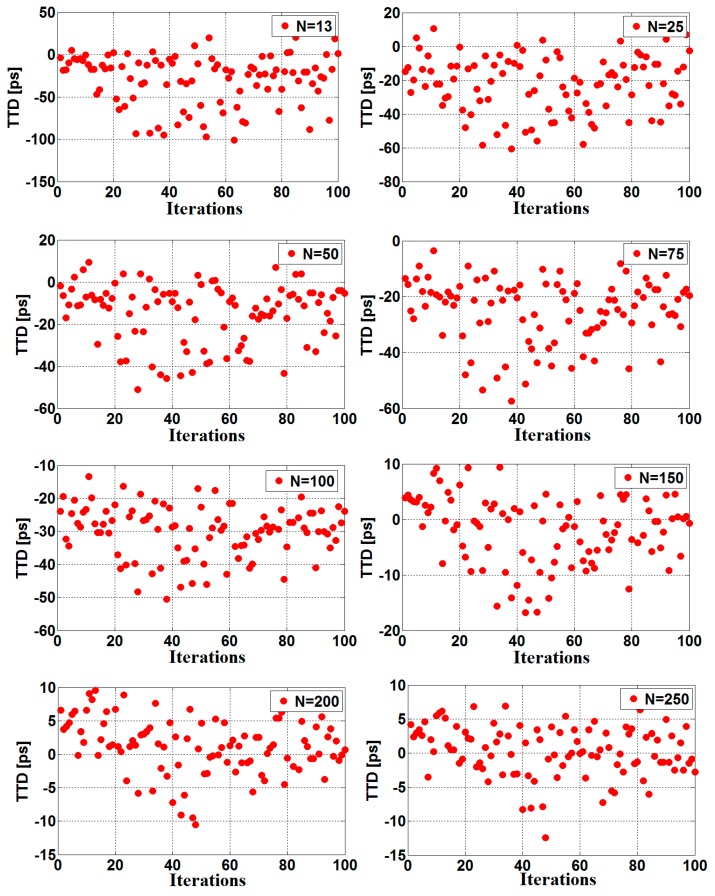
TTD results measured for different number of fitted samples (N).

**Figure 9:**
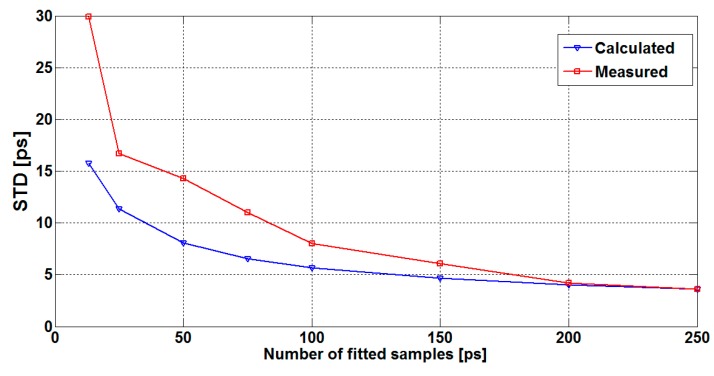
TTD standard deviation versus the number of fitted samples (N).

As mentioned previously, the jitter can also be reduced through increasing the number of the signal samples, used to adjust the sine-fitting parameters. Since the noise present at each sampling sequence is uncorrelated, a given number of samples N reduces the timing jitter values by a factor of $\sqrt{N}$ (according to the averaging principle). Figure 8 depicts a single TTD measurement carried out at room temperature and no-flow conditions for different numbers of the fitted samples. Figure 9 illustrates the measured TTD standard deviation (STD) versus the number of fitted samples, as well as the theoretical limits estimated using Equation (15). Based on these results, it can be deduced that the measured jitter exhibits the same behavior as the calculated one. In other words, as the number of samples increases, the standard deviation of the measured TTD decreases, according to Equation (15).

(15) $$\sigma_{\overset{\prime }{x}}=\frac{\sigma }{\sqrt{N}}$$

## 6. Zero Flow TTD-Offset Correction

**Figure 10:**
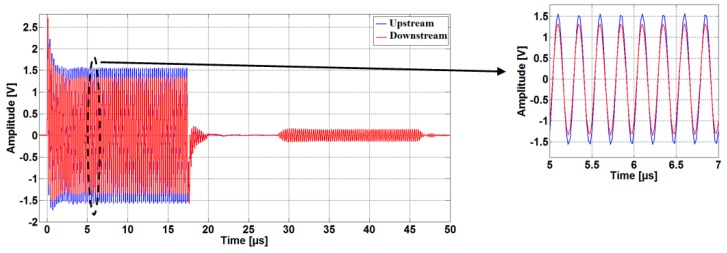
Acquired ultrasonic signals in the upstream and downstream directions (measured using a driving frequency of 4 MHz).

As mentioned before, in order to eliminate the possibility that the meter detects a false flow under no-flow conditions, the upstream and downstream transit times should be ideally the same, but this may not be the case unless special precautions are taken. Due to the fact that every flow direction exhibits a slightly different electrical impedance compared to the other, this leads to a dissymmetry between upstream and downstream signal paths (in term of the electrical impedances of the electronics and the transducers employed in the meter) [15] and causing different amplitudes in the two upstream and downstream transmitted signals. Figure 10 shows a measurement results carried out at room temperature and no-flow conditions. Both transducers are excited with the same sinus burst at a 4 MHz forced frequency. The 250 mV transmitter amplitudes difference seen in Figure 10 results in about a 150 ps zero-flow TTD-offset.

Irrespective of the mismatch of the transducers, the variation of the resonance frequency with the operating temperature range [6,16,17,18] can cause a dissymmetry between the two directions of the ultrasonic signal paths. Thus, the temperature variation of the medium is the main reason for the zero-flow TTD-offset drift.

In order to effectively eliminate TTD-offset at no-flow conditions, a matching between the upstream and downstream electrical impedance of both transducers and their associated electronic is required to reach a highly symmetrical signal path. According to the literature [19], the electrical impedance of a transducer can be controlled by the forced frequency. We have used this feature to eliminate the electrical impedance mismatch between both directions, because a well-matched upstream downstream signal path reduces the transmitter amplitudes difference and results in a very small zero-flow TTD-offset.

**Figure 11:**
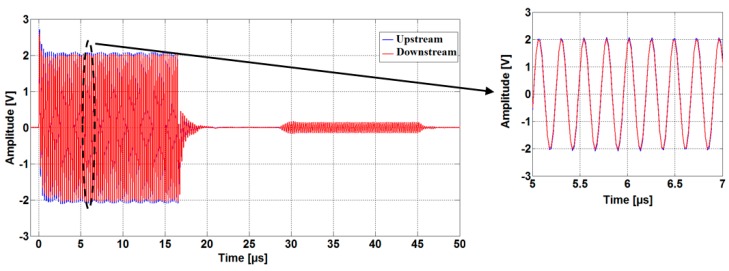
Acquired ultrasonic signals in the upstream and downstream directions (measured using a driving frequency of 4.19 MHz).

By changing the sinus burst frequency from 4 MHz to 4.19 MHz, the 150 ps zero-flow TTD-offset measured previously is reduced substantially to less than 5 ps (Figure 11). Moreover, comparing the measured transmitted signals depicted in Figure 10 and Figure 11, it can be observed that the difference between the two amplitudes is reduced from 250 mV measured at 4 MHz to less than 15 mV achieved by exciting the transducers at 4.19 MHz forced frequency under the same previously mentioned conditions.

**Figure 12:**
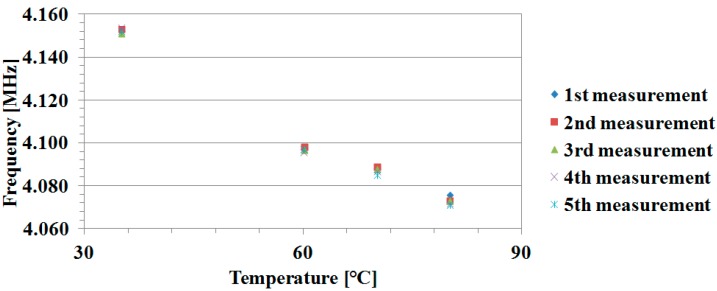
Temperature dependency of the forced frequency used for the zero-offset calibration.

As a summary, choosing the correct forced frequency can drastically reduce the zero-offset error. To realize an automatic compensation, one needs to accurately set the forced frequency suitable to eliminate the offset caused by the temperature variation inside the pipe. In order to ensure the repeatability or the precision of the driving frequency used to compensate the zero-flow offset, several experimental measurements have been performed at four randomly chosen different temperatures (35 °C, 60 °C, 70 °C, and 80 °C). These experiments lead to the results illustrated in Figure 12. Therefore, we have shown that there is a clear, simple, stable, and reliable relation between the needed excitation forced frequency and temperature.

## 7. TTD Measurement Results and Analysis

The measurement flow meter pipe used in the experiments is a D-Flow pipe provided by Ultrasonic Technology of Sweden (Figure 1), with a length L = 42.2 mm and an inner diameter is 8 mm. The piezo-based transducers are made of polyetheretherketone (PEEK).

**Figure 13:**
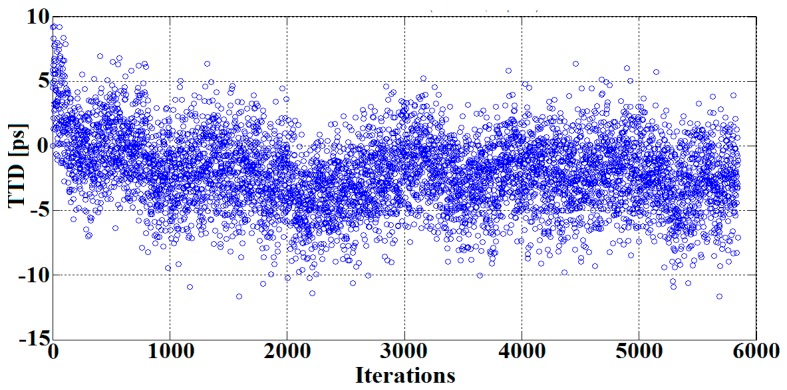
Calibrated zero-flow TTD-offset measured results at 80 °C.

The TTD value is evaluated based on 250 samples taken from the last 20 cycles of the steady-state region for both received signals. For each measurement setup, the TTD is computed several times with a repetition rate of about 1.6 s. In order to emulate the operation temperature from around 25 °C to 80 °C, the flow meter pipe is put in a water bath, with a thermostat to regulate the temperature around the transducers. The temperature of the transducer can be considered the same as the still water filled inside the pipe. Figure 13 illustrates the TTD measurement results obtained at 80 °C fixed temperature. Based on this result, the measured jitter is in the range of 4 ps (evaluated as a standard deviation). With respect to 80 °C, the zero-flow TTD-offset adjustment was reached by tuning the forced frequency of the exciting burst to 4.075 MHz.

**Figure 14:**
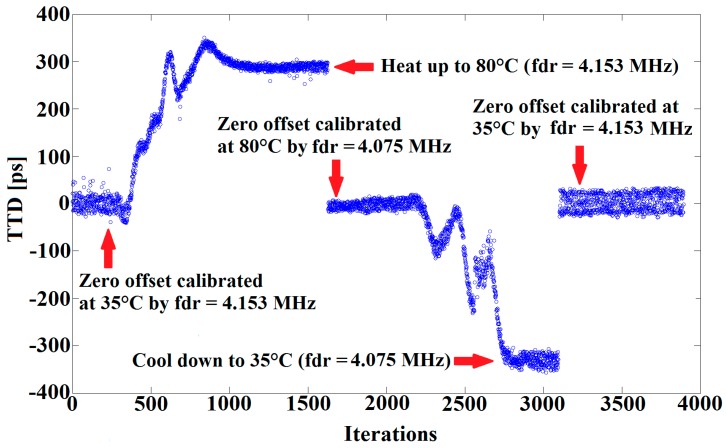
TTD measurements performed over a temperature range from 35 °C to 80 °C.

Figure 14 provides the results for zero-flow TTD-offset calibration, using an appropriate forced frequency with respect to medium temperature. The results below show a reduction of the TTD-offset to almost zero over a temperature range from 35 °C to 80 °C. This is achieved by optimizing the impedance matching of both upstream and downstream signal paths using the correct forced frequency.

## 8. Conclusions

The assumption established throughout this work is that the proposed jitter and zero-flow TTD reduction approaches considerably enhance the measurement performances. As it can be inferred from the experimental results, the standard deviation of the jitter is reduced drastically through using the sine-fitting technique. Furthermore, the system accuracy is carried out one step further by exploring two techniques, namely, using low noise amplifiers to improve the system dynamic range and an adequate number of samples in a specific time interval (steady-state region). Moreover, the present work proposed a new improved method to eliminate the zero-flow TTD-offset and TTD-offset drift. This method is based on the principle that continuously adjusting the forced frequency nearby the resonance working area of the transducers according to a specific strategy reduces the zero-flow TTD-offset to zero.
